# Facial Expression Recognition with Fusion Features Extracted from Salient Facial Areas

**DOI:** 10.3390/s17040712

**Published:** 2017-03-29

**Authors:** Yanpeng Liu, Yibin Li, Xin Ma, Rui Song

**Affiliations:** School of Control Science and Engineering, Shandong University, Jinan 250061, China; liuyanpeng@sucro.org (Y.L.); liyb@sdu.edu.cn (Y.L.); maxin@sdu.edu.cn (X.M.)

**Keywords:** facial expression recognition, fusion features, salient facial areas, hand-crafted features, feature correction

## Abstract

In the pattern recognition domain, deep architectures are currently widely used and they have achieved fine results. However, these deep architectures make particular demands, especially in terms of their requirement for big datasets and GPU. Aiming to gain better results without deep networks, we propose a simplified algorithm framework using fusion features extracted from the salient areas of faces. Furthermore, the proposed algorithm has achieved a better result than some deep architectures. For extracting more effective features, this paper firstly defines the salient areas on the faces. This paper normalizes the salient areas of the same location in the faces to the same size; therefore, it can extracts more similar features from different subjects. LBP and HOG features are extracted from the salient areas, fusion features’ dimensions are reduced by Principal Component Analysis (PCA) and we apply several classifiers to classify the six basic expressions at once. This paper proposes a salient areas definitude method which uses peak expressions frames compared with neutral faces. This paper also proposes and applies the idea of normalizing the salient areas to align the specific areas which express the different expressions. As a result, the salient areas found from different subjects are the same size. In addition, the gamma correction method is firstly applied on LBP features in our algorithm framework which improves our recognition rates significantly. By applying this algorithm framework, our research has gained state-of-the-art performances on CK+ database and JAFFE database.

## 1. Introduction

Facial expression plays an important role in our daily communication with other people. For the development of intelligent robots, especially indoor mobile robots, emotional interactions between robots and humans are the foundational functions of these intelligent robots. With automated facial expression recognition technology, these home service robots can talk to children and take care of older generations. Also, this technology can help doctors to monitor patients, which will save hospitals much time and money. In addition, facial expression technology can be applied in a car to identify whether the driver has fatigue, and this can save many lives. Facial expression recognition is worth researching because many situations need this technology. Many research works have been done in the literature; the universal expressions mentioned in papers are usually: anger, disgust, fear, happiness, sadness and surprise [1,2,3] while some researchers add neutral and contempt [4,5]. Different sensors are used to capture data of these expressions, and researchers recognize these basic expressions from two-dimensional (2D) and 3D spaces [6,7] faces. While different methods are applied to recognize the basic expressions in 2D and 3D spaces, landmarks localization processes are used in both 2D and 3D data. Vezzetti et al. [8,9] extracted many landmarks from multiexpression faces relying on facial geometrical properties, which makes it easy to localize these parts on 3D faces. Many application scenarios, such as service robots, apply 2D images to detect and recognize faces, so our research focuses on recognizing expressions from 2D static images.

Different styles of data and various frameworks are applied to 2D space facial expressions recognition. Like other recognition research, facial expressions recognition uses data from videos, images sequences [10] and static images [3,11]. All of the movement processes of the expressions are applied in the research, which use videos and images. Research using static images only uses the peak frames because they contain sufficient information about the specific expressions, and that is also the reason why this paper chose to use the peak frames. There are two main kinds of algorithm frameworks applied in facial expressions recognition work. Algorithms that use mature descriptors such as Histogram of Oriented Gradient (HOG) [12] and Local Binary Patterns (LBP) [4] extract features from the images and then send the features to the classifiers. The performances of this kind of algorithm framework rely on the effectiveness of these descriptors. In order to fuse more effective descriptors, researchers extract different kinds of features and fuse them together [13]. Although the fusion features behave better than one kind of feature, these features’ distinguishing features have not been fully used. Feature correction method is applied to the features in our paper and this significantly improves the recognition rate. Deep networks is another popular framework in the facial expression recognition domain. AU-inspired Deep Networks (AUDN) [2], Deep Belief Networks (DBN) [14] and the Convolutional Neural Network (CNN) [10,15] are used in facial expressions recognition work. Apart from the higher recognition rate, more computing resources and data are needed in these algorithms. For these reasons, the former framework is applied in our research.

Face alignment is applied to help researchers to extract more effective features from the static images [4,16]. Automated facial landmark detection is the first step to complete this work. After finding these landmarks on the faces, researchers can align the faces and extract features from these faces. Early days, for the limitation of face alignment technology, researchers use fewer landmarks to align the faces and separate the faces to several small patches for extracting features [13]. This can roughly align the faces while more landmarks can improve the alignment precision. There are many methods to detect landmarks from the faces. Tzimiropoulos et al. [17] proposed a Fast-SIC method for fitting AAMs to detect marks on the faces. Zhu et al. [18] use a model based on the mixture of trees with a shared pool which marks 68 landmarks on the face. This method is applied in our algorithm and 68 landmarks are used to align the salient areas. These landmarks mark the shape of the eyebrows, eyes, nose, mouth and the whole face, which can help researchers to cut the salient patches. Although alignment faces can help to extract more effective features from the faces, some areas on the faces do not align well during this process. In this paper, the idea of normalizing the salient areas is firstly proposed to improve the features’ extracted effectiveness.

In order to reduce the features’ dimensions and extract more effective features, different salient areas definitude methods are proposed in the literature. Zhong et al. [3] explained the idea that discovering common patches across all the expressions is actually equivalent to learning the shared discriminative patches for all the expressions in their paper. They transferred the problem into a multi-task sparse learning (MTSL) problem and by using this method they obtained a good result. Happy et al. [13] applied these areas found in their paper and they also gained a decent result. Liu et al. [2] used Micro-Action-Pattern Representation in the AU-inspired deep networks (AUDN) and built four criterions to construct the receptive fields. This gained a better result and accomplished feature extraction at the same time. In order to define the salient areas more accurately, our research uses neutral faces to compare with the peak frames of these expressions. The Karl Pearson correlation coefficient [19] is applied to evaluate the correlation between the neutral faces and the faces that expressed different expressions. For finding the precise locations of the salient areas, the faces are separated to several small patches. After comparing the small patches to their neutral faces, the patches which have weaker correlation coefficient are found and these are the areas expressing the specific expression. By using this method, the salient areas of the six fundamental expressions are found and after fusing these areas, the salient areas of the six basic expressions are found too. Landmarks of the faces are used to locate these salient areas and different sizes of salient areas are normalized in our research framework.

Different kinds of descriptors are applied in facial expressions recognition research. Regarding the scale of the features extracted areas, previously, hand-crafted features were extracted from the whole alignment face [4,16] but nowadays the salient areas are used in hand-crafted extraction [3,13]. Aiming to describe the different expressions more effectively, diverse features extracted methods are used in facial expression recognition. Typical hand-crafted features include Local Binary Patterns (LBP) [4], Histogram of Oriented Gradient (HOG) [12], Scale Invariant Feature Transform (SIFT) [20], and the fusion of these features [11]. According to the literature, the fusion features contain more information about these expressions and achieve better results. That is the reason why we chose to extract LBP and HOG from the faces. Although fusion features can improve recognition rate, it is hard to fuse these features well. Before different features fuse together, normalization methods are applied to the features. Although utilizing this normalization method can improve the recognition result, different kinds of features’ identities cannot mix well. Aiming to use more information of the LBP features and normalize the LBP features, the gamma feature correction method is firstly applied on LBP features in our algorithm framework.

**Figure 1:**
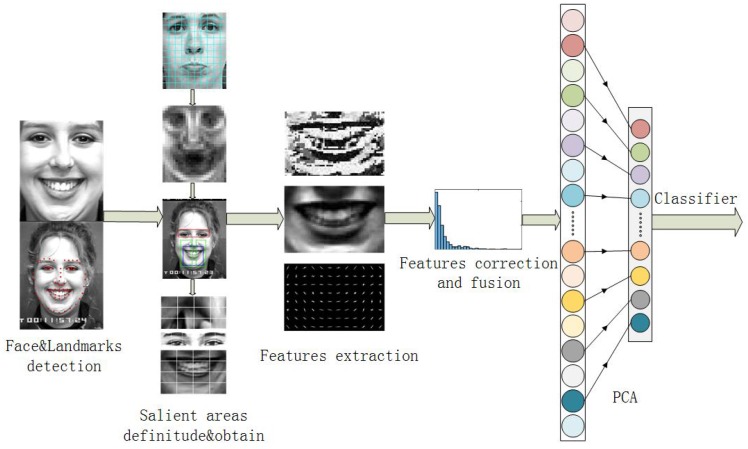
Framework of the proposed algorithm.

In this paper, a straightforward but effective algorithm framework has been proposed to recognize the six basic expressions from static images. The algorithm framework is shown in Figure 1. In order to define and obtain these salient areas from these faces, the faces and facial landmarks are detected in the first step. After doing that, these faces are separated into several patches and by comparing neutral faces to these expressions, the salient areas are defined. Until this step, the salient areas are separated from the faces according to these landmarks. For extracting more effective features from these salient areas, the idea of normalizing the salient areas is firstly proposed to overcome salient areas misalignment. After finishing that, LBP and HOG features are extracted from these salient areas. The gamma correction method is firstly applied on LBP features and then the classifier can use more information from these LBP features. The Z-score method is used to normalize the LBP and HOG features to fusing them. Before applying different classifiers to classify these six expressions, Principal Component Analysis (PCA) is utilized to reduce the dimensions. Finally, different classifiers are applied to evaluate the effect of our framework, and our framework has achieved a better grade than the deep networks [2,14].

## 2. Methodology

In this section, the proposed algorithm will be explained in detail. This section will introduce the salient facial areas definitude principle and show the salient areas normalization and features fusion methods. LBP features correction methods will be introduced and applied in our algorithm. We will then introduce the following sections of this paper.

### 2.1. Faces Alignment and Salient Facial Areas Definitude

Automated face and facial landmark detection is the first step in our method. Facial landmark detection is an important base for facial expression classification. The method that is applied in the paper [18] is chosen to mark 68 landmarks on faces in our research. These landmarks mark the shape of the eyebrows, eyes, nose, mouth and the whole face, so these specific areas can be located by these landmarks. These 68 landmarks on the face and the normalized face have been shown in Figure 1. According to the average length and width of the faces and the proportion of the length and width, the faces in CK+ database are normalized to 240 × 200.

As we all know, these six fundamental expressions have different salient areas. In this paper, an algorithm is proposed to find the salient areas in these expressions. For the purpose of extracting more effective features from the faces, people have applied different methods to calculate the salient areas in the faces. Zhong et al. [3] explained the idea that discovering the common patches across all the expressions is actually equivalent to learning the shared discriminative patches for all the expressions in the paper. Since multi-task sparse learning (MTSL) can learn common representations among multiple related tasks [21], they transferred the problem into an MTSL problem. They used this method and gained a good result. In order to learn expression specific features automatically, Liu et al. [2] proposed an AU-inspired deep network (AUDN). They used Micro-Action-Pattern Representation in the AUDN and built four criterions to construct the receptive fields. This gained a better result and accomplished features extraction at the same time.

These methods all found salient areas from the aligned faces and extracted features from these salient areas. As for our algorithm, the areas which are more salient to their own neutral faces are firstly found. In the last paragraph, the 68 landmarks have been found and the faces are normalized to 240 × 200. The areas in the six basic expressions are compared to their neutral faces at the same location. If the areas during the expressions have not moved around, the areas must have more correlation with the areas on the neutral faces. Using this principle, compared to the other correlation coefficient methods in [19] the Karl Pearson correlation coefficient is applied to evaluate the correlation between the neutral faces and the faces expressed in different expressions. The Karl Pearson correlation coefficient is applied to evaluate the correlation between the matrixes. For finding the precise locations of the salient areas, the faces are separated to 750 (30 × 25) patches and every patch is 8 × 8 pixels. These 8 × 8 pixels patches are matrixes and by comparing the small patches from neutral faces and specific expressions, the salient areas can be precisely found. The Karl Pearson correlation coefficient formulate is shown next.

(1) $$\gamma_{ij}^{k}=\frac{\sum_{m}\left(E_{m}-\bar{E}\right)\left(N_{m}-\bar{N}\right)}{\sqrt{\left(\sum_{m}{\left(E_{m}-\bar{E}\right)}^{2}\right)\left(\sum_{m}{\left(N_{m}-\bar{N}\right)}^{2}\right)}}$$

where $\gamma_{ij}^{k}$ is the (i,j)th patch’s correlation coefficient of specific expressions, so the scale of *i* is 1 to 30, *j* is 1 to 25. $E_{m}$ is the pixel value of one subject from the specific expression and *m* ranges from 1 to 64 while *N* is the pixel value of the neutral face of that subject. $\bar{E}$ is the mean of the small patch from specific expression, and $\bar{N}$ is the mean of the neutral face.

(2) $$R_{ij}=\sum_{k}\rho_{k}*\gamma_{ij}^{k}$$

In order to find the salient areas of all these six basic expressions, a formula is defined to evaluate the final correlation coefficient. The $R_{ij}$ is the final correlation coefficient of the (i,j)th location on face. By changing the $\rho_{k}$, the different proportion of the *k*th expression can be changed. Besides, the sum of the $\rho_{k}$ must equal 1.

(3) $$\sum_{k}^{6}\rho_{k}=1$$

**Figure 2:**
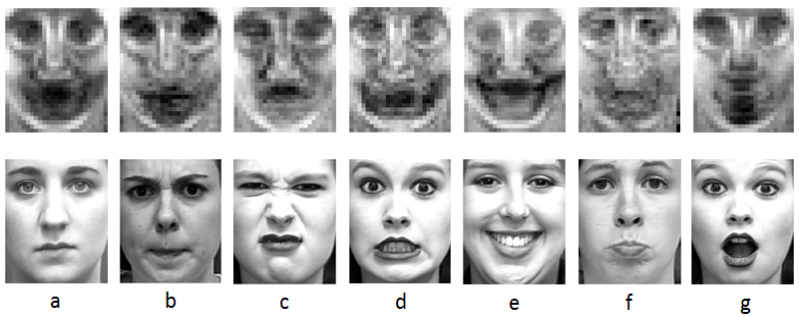
The salient areas of the six expressions. (**a**) Salient areas of all expressions and neutral; (**b**) Salient areas of anger; (**c**) Salient areas of disgust; (**d**) Salient areas of fear; (**e**) Salient areas of happy; (**f**) Salient areas of sad; (**g**) Salient areas of surprise.

The results of the six expressions are shown in Figure 2. The areas found in this section will be applied in the next section. From Figure 2, we can find that different expressions have different salient areas. Equation (1) is used to evaluate the salient areas in the six fundamental expressions. In Equation (3), $\rho_{k}$ expresses the proportion of the specific expression in the final result, the value of $\rho_{k}$ can be changed according to the numbers of these different expressions because there are different numbers of images in these expressions.

### 2.2. Salient Areas Normalization and Features Extraction

In this section, the idea of normalizing the salient areas rather than the whole faces is proposed and applied. Furthermore, local binary patterns (LBP) features and the histogram of oriented gradient (HOG) features all are extracted from the salient areas. Compared to the method extracting features from the whole faces, features extracted from salient areas can reduce the dimensions, lower noise impacts and avoid overfitting.

#### 2.2.1. Salient Areas Normalization

In the last section, these salient areas are determined. Our research has a similar result as the result in [3], but the performance differs in the eye areas. In papers [3,13], the researchers used the patches of the faces which come from alignment faces. Normalizing the whole faces is a good idea before more landmarks are marked from the faces. Then, more landmarks can be marked from the faces, which makes it easier to extract the salient areas from the faces. There are two main reasons for choosing to normalize the salient areas.

Firstly, aligning the faces may result in salient areas being misaligned. In order to demonstrate the alignment effects, the faces are aligned and then all the faces in one specific expression are added to gain the average face. The each pixel $X_{ij}$ is the average of all images of the specific expression.

**Figure 3:**
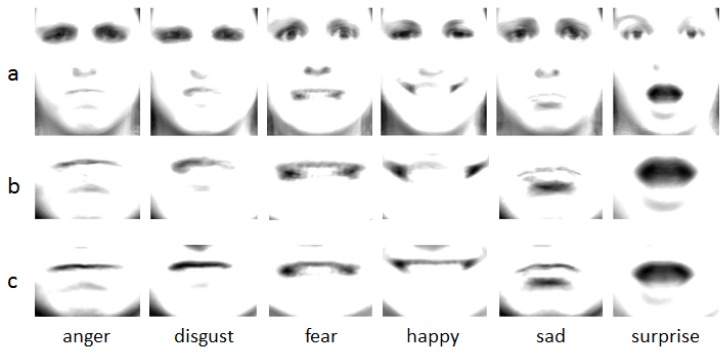
Average faces and the average salient areas. (**a**) Average faces of the six expressions; (**b**) Mouth areas of the average faces; (**c**) Average salient mouth areas.

The salient mouth parts are separated from the faces and the average salient mouth areas are calculated for comparison. Figure 3 shows the result of the average faces, average salient areas and the mouth parts of the average faces. The mouth parts of the average faces are used to compare with the mouth parts using salient areas alignment. From the figure, it is clear that the mouth parts of the average faces have weaker contrast than the alignment mouth parts. This explains that aligning the faces leads to salient areas being misaligned. In contrast, by aligning the salient mouth areas, the mouths have a clear outline. Moreover, the alignment faces have different sized salient areas. Different faces have different size, and the different sizes of the salient areas are extracted from these faces when we just use these landmarks to cut these salient areas. In the end, different dimensions of LBP and HOG are extracted from these salient areas. Using these features to classify the expressions can lead to worse recognition result. The reason why different LBP and HOG dimensions are extracted from these different sizes of salient areas can be explained by the principles of LBP and HOG which will be introduced in the next part. Aligning the whole faces may obtain different features from the same expression subjects because they have different sized areas to express the expression. This negatively influences the feature training in our algorithm framework. These are the reasons why the salient areas are normalized in our algorithm. Because our salient areas normalization method can overcome these shortcomings, our algorithm can gain a better result than only using the face alignment methods. In the experimental section, comparative experiments will be designed to compare the effects of the salient areas alignment method with the traditional faces alignment methods.

(4) $$X_{ij}=\frac{1}{n}\sum x_{ij}$$

#### 2.2.2. Features Extraction

●  Local Binary Patterns (LBP)

**Figure 4:**
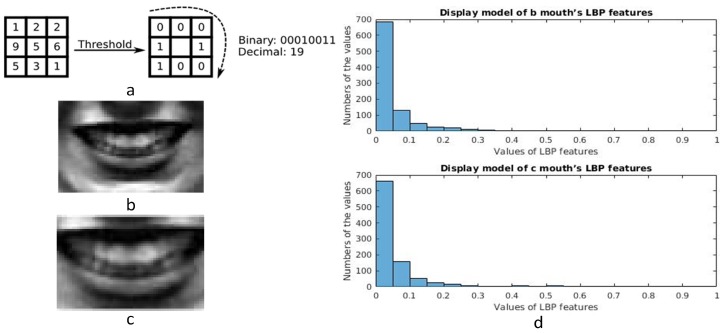
(**a**) Calculation progress of the original Local Binary Patterns (LBP) value; (**b**) Mouth area with pixels of 40 × 60; (**c**) Mouth area with the former pixels of 28 × 42, the real pixels are enlarged from the former image; (**d**) Display models of (**b**,**c**) mouth.

**Figure 5:**
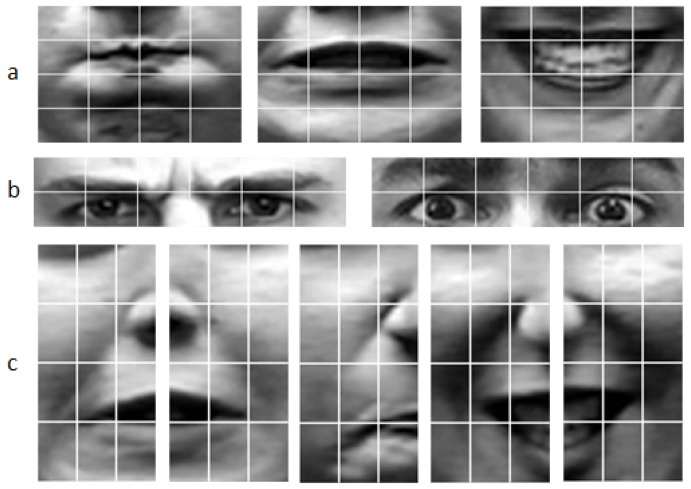
Small patches of salient areas. (**a**) Mouth areas, anger, fear, happy; (**b**) Forehead areas, anger, fear; (**c**) Cheek areas, left cheek fear, right cheek fear, left cheek anger, left cheek happy, right cheek happy.

Texture information is an important descriptor for pattern analysis of images, and local binary patterns (LBP) were presented to gain texture information from the images. LBP was first described in 1994 [22,23] and from then on LBP has been found to be a powerful feature for texture representation. As for these facial expressions, actions of the muscles on the faces lead the faces to generate different textures. LBP features can describe the texture information of the images and this is the reason why LBP features are extracted from the salient areas. The calculation progress of the original LBP value is shown in Figure 4a. A useful extension to the original operator is the so-called uniform pattern [24], which can be used to reduce the length of the feature vector and implement a simple rotation invariant descriptor. In our research, a uniform pattern LBP descriptor is applied to gain features from the salient areas, and the salient areas are all separated to small patches. LBP features are gained from these salient areas respectively and these features are concatenated as the final LBP features. The length of the feature vector for a single cell can be reduced from 256 to 59 by using uniform patterns. This is very important, because there are many small patches in our algorithm. For example, the size of the mouth area is 40 × 60 and the small patches’ size is 10 × 15, so the mouth area is divided into 16(4 × 4) patches. The uniform LBP features are extracted from each small patch and mapped to a 59-dimensional histogram. The salient areas all are separated into several small patches and the results are shown in Figure 5. The numbers are shown in Table 1. The dimension of the final LBP features is found to be 3068 by adding the numbers in Table 1.

Becuase different features will be extracted from different sizes of salient areas, these salient areas should be aligned. In order to demonstrate the difference, different sizes of mouth areas are cut from one face and these areas are normalized to the same size. These mouth areas are shown in Figure 4. LBP features are extracted from these normalized faces and their distributions are shown in Figure 4. From the figure, we can know that the values in (d) and (e) result in different performance, and a conclusion can be drawn that different sizes of images have different LBP features. Besides, HOG features also are extracted from these salient areas, and they have a similar result as for LBP features.

●  Histogram of Oriented Gradient (HOG)

**Figure 6:**
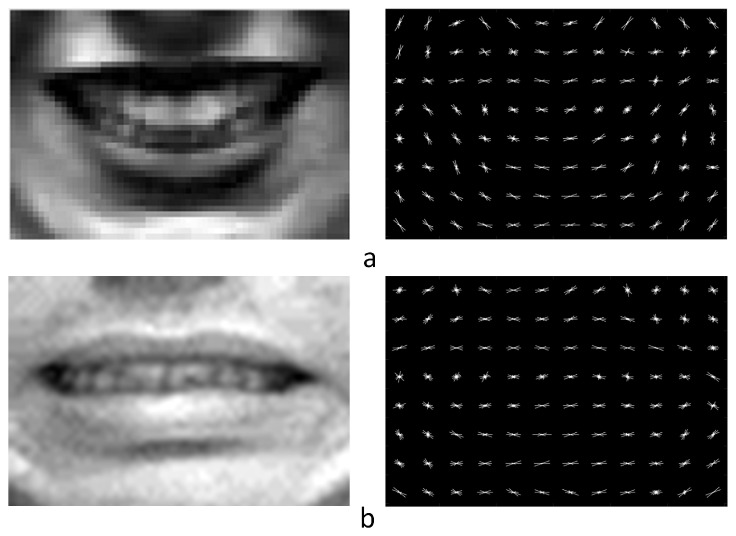
HOG descriptors of the mouths. (**a**) Happy mouth areas; (**b**) Fear mouth areas.

Histogram of oriented gradients (HOG) is a feature descriptor which is used in computer vision and image processing [25]. The technique counts occurrences of gradient orientation in localized portions of an image. HOG descriptors were first described in 2005 [26], the writers used HOG for pedestrian detection in static images. During HOG features extraction, the image is divided into several blocks and the histograms of different edges are concatenated as shape descriptor. HOG is invariant to geometric and photometric transformations, except for object orientation. Because the images that in these databases have different light conditions and different expressions have different orientations in the eyes, nose, lips corners, as a powerful descriptor HOG is selected in our algorithm. In our paper, for extracting HOG features, every cell is 5 × 5 and 4(2 × 2) cells make up a patch. The dimension of the mouth area is 60 × 40 and every cell has 9 features, so the dimension of the mouth area is 2772. The dimensions of the four salient areas are shown in Table 2. The HOG descriptors are shown in Figure 6 and the figure shows that the mouth areas of different expression have different HOG descriptors.

### 2.3. Features Correction and Features Fusion

#### 2.3.1. LBP Correction

LBP feature is a very effective descriptor of the texture information of images. Many researchers [3,13] applied LBP to describe these different expressions. In our algorithm, LBP features are extracted and processed before they are sent into the classifiers. For most papers, researchers extract features from images and normalize the data to 0–1, e.g., [27] normalizes the data to 0–1. For our algorithm framework, the image’s data are normalized to 0–1, and by using this method a better recognition rate can be gained. In order to normalize the LBP features of every subject, the method in Equation (5) is applied to normalize the LBP features to 0–1.

(5) $$L_{m}=\frac{l_{m}}{max(l_{m})}$$

where *m* is the dimension number of every salient area and $l_{m}$ is the value of the LBP feature.

**Figure 7:**
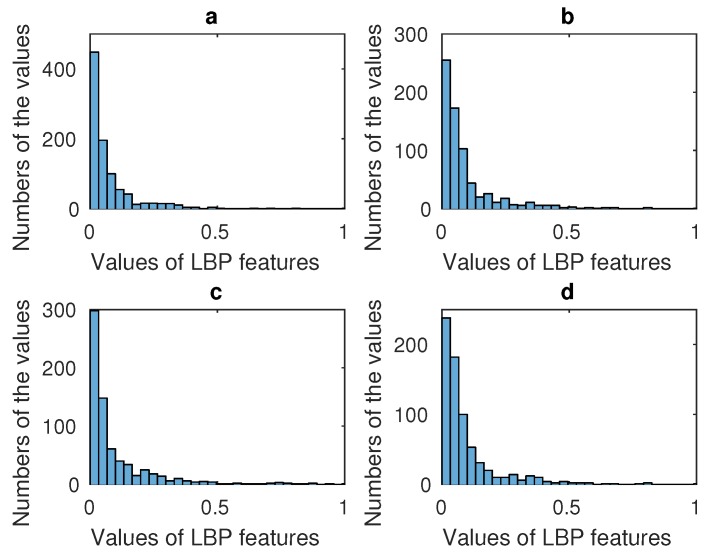
(**a**) Display model of the mouth’s LBP features; (**b**) Display model of the left cheek’s LBP features; (**c**) Display model of forehead’s LBP features; (**d**) Display model of the right cheek’s LBP features.

Aiming to utilize the specific characteristic of every area, these four salient areas are normalized respectively. The distribution styles of these LBP features extracted from the four salient areas are displayed in Figure 7. The figure shows that the distribution model of LBP features is the power law distribution. In image processing, the gamma correction redistributes native camera tonal levels into ones which are more perceptually uniform, thereby making the most efficient use of a given bit depth. In our algorithm, the distribution of the LBP features is the power law distribution, and therefore more information concentrates on minority LBP features. According to our experiment, although the distributions of these subjects’ LBP features all follow the power law distribution, the specific values are slightly different. For example, LBP values from 0 to 0.5 have different numbers when different subjects’ images are processed. This can be changed by gamma correction method, and by doing this, more information can concentrate on more LBP features. For using more information from LBP features and making it easy to fuse LBP and HOG features, gamma correction is used to correct the LBP feature data.

(6) $${\bar{L}}_{m}=L_{m}^{\frac{1}{\lambda }}$$

where λ is the correct gamma number. As is well known, most values of the gamma number come from experimental experience data. The parameter σ is proposed in our algorithm to help to find the proper λ. In Equation (7) we have defined the mathematical expression of σ. From Equation (7) we know that σ has a similar meaning to variance and the zero value is separated from these data because it has no meaning. The gamma correction method is proposed to use more information from the initial LBP features, and the value of variance shows the fluctuation of the data. For the power law distribution, fewer data contain more information. As for the LBP features when the gamma correction method is applied, more LBP features contain more information and therefore the fluctuation of LBP features becomes bigger. That is the reason why parameter σ is proposed and applied, and the relationship between σ and the gamma number λ proves the correctness of our method.

**Figure 8:**
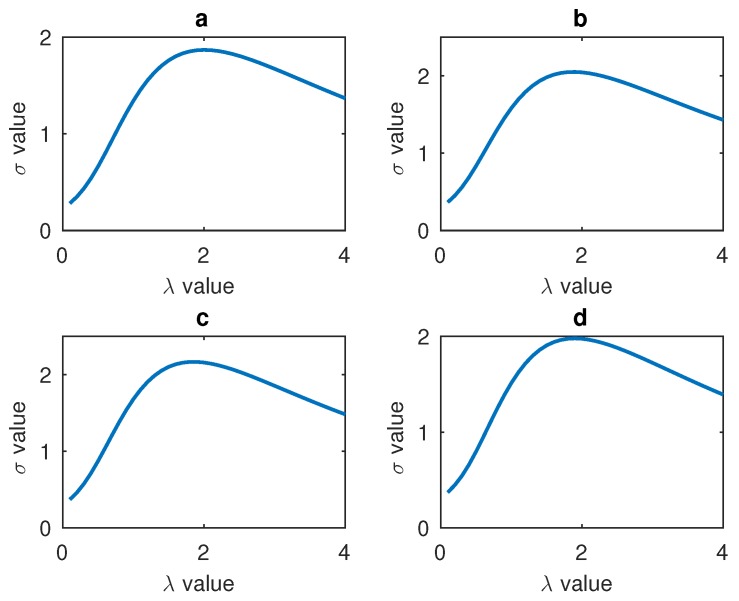
(**a**) Relationship between the mouth’s λ and σ; (**b**) Relationship between the left cheek’s λ and σ; (**c**) Relationship between the forehead’s λ and σ; (**d**) Relationship between the right cheek’s λ and σ.

**Figure 9:**
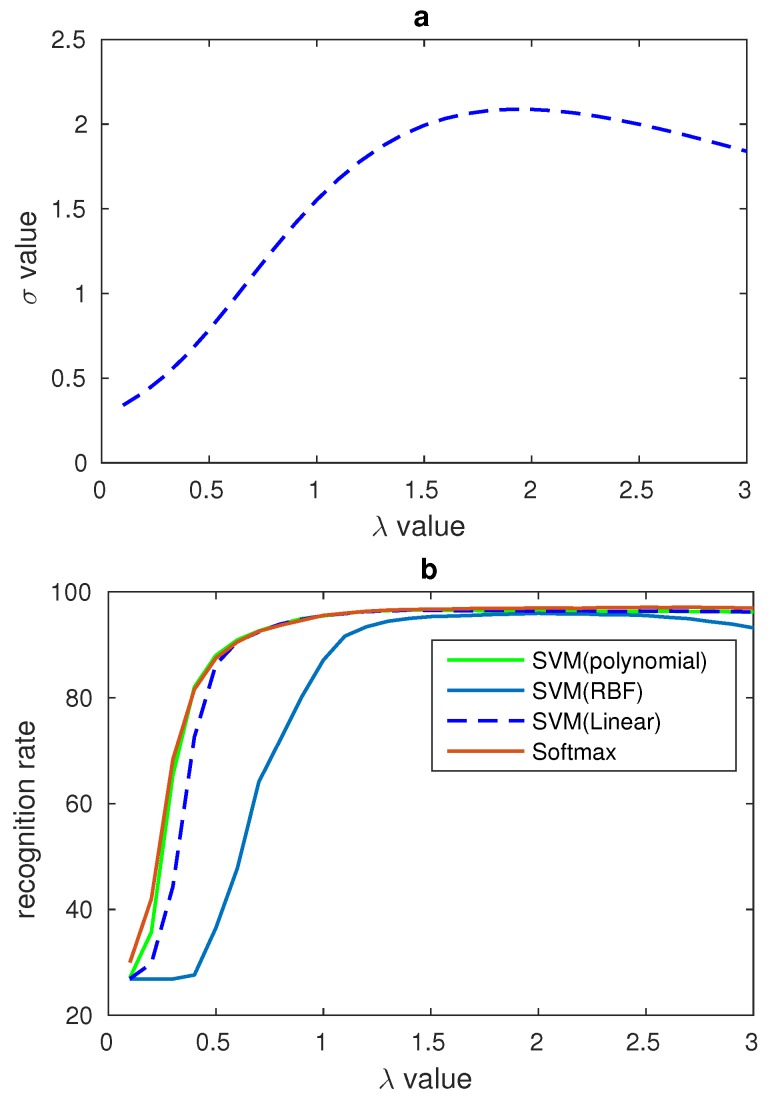
(**a**) Relationship between λ and σ on CK+ database; (**b**) Relationship between λ and recognition rates from different classifiers on CK+ database.

Every salient area is processed respectively, so four σ values will be gained. The relationships between these four salient areas’ σ and the gamma number λ are shown in Figure 8. According to the experimental data, all the four salient areas have the maximum σ value around λ’s value 2. Therefore, all the four salient areas’ σ values are added to find the final sum and the related λ.

(7) $$\sigma =\frac{1}{np}\sum_{n}\sum_{p}{\left(L_{np}-U_{n}\right)}^{2}$$

(8) $$U_{n}=\frac{1}{p}\sum_{p}L_{np}$$

where *n* is the number of the images’ number, and *p* is the number of nonzero data in LBP features of the salient areas. Therefore, *p* is smaller than the dimensions of the salient areas’ LBP features. $L_{n}p$ is the value of LBP feature and $U_{n}$ is the mean value of LBP features from the specific subjects. In our algorithm, the relationship between the σ, gamma number λ and the recognition rate have been found, and the relationship is shown in Figure 9.

#### 2.3.2. HOG Processing and Features Fusion

Different features describe different characters of the images; therefore, researchers have merged some features together to be able to take advantage of the superiority of all the features [13,27]. For our algorithm, the LBP and HOG descriptors are applied to utilize the texture and orientation information of these expressions. Proper fusion methods are very important factors for recognition work and unsuitable methods can make the recognition result worse. The recognition rate of the individual feature and fusion features will be shown in the experimental section. Zhang et al. [27] applied a structured regularization(SR) method which is employed to enforce and learn the modality specific sparsity and density of each modality, respectively. As for our algorithm, the single features are firstly processed to their best performance and then they are normalized to the same scale. In the experimental section, different experiments are proposed to explain the results of single features and the fusion feature.

The gamma correction method is applied to ensure the LBP achieve their best performance. Different features must be processed to the same scale when these features are fused together. The Z-score method is used to process LBP and HOG features, and after applying the method the average is 0 while the variance is 1.

(9) $$\sigma =\sum_{j}^{J}{\left(f_{j}-\mu \right)}^{2}$$

(10) $$\mu =\frac{\sum_{j}^{J}f_{j}}{J}$$

(11) $${\hat{f}}_{j}=K\frac{\left(x_{j}-\mu \right)}{\sigma +C}$$

where $f_{j}$ is the data of LBP or HOG feature and ${\hat{f}}_{j}$ is the feature data after processing. As for the LBP features, although the display model has changed, the data are changed to the same scale and a better result can be gained. Because ${\hat{f}}_{j}$ is too small, number *K* is used to multiply ${\hat{f}}_{j}$. In our experiment, the *K* equal to 100.

### 2.4. Principal Component Analysis( PCA) and Classifiers

**Figure 10:**
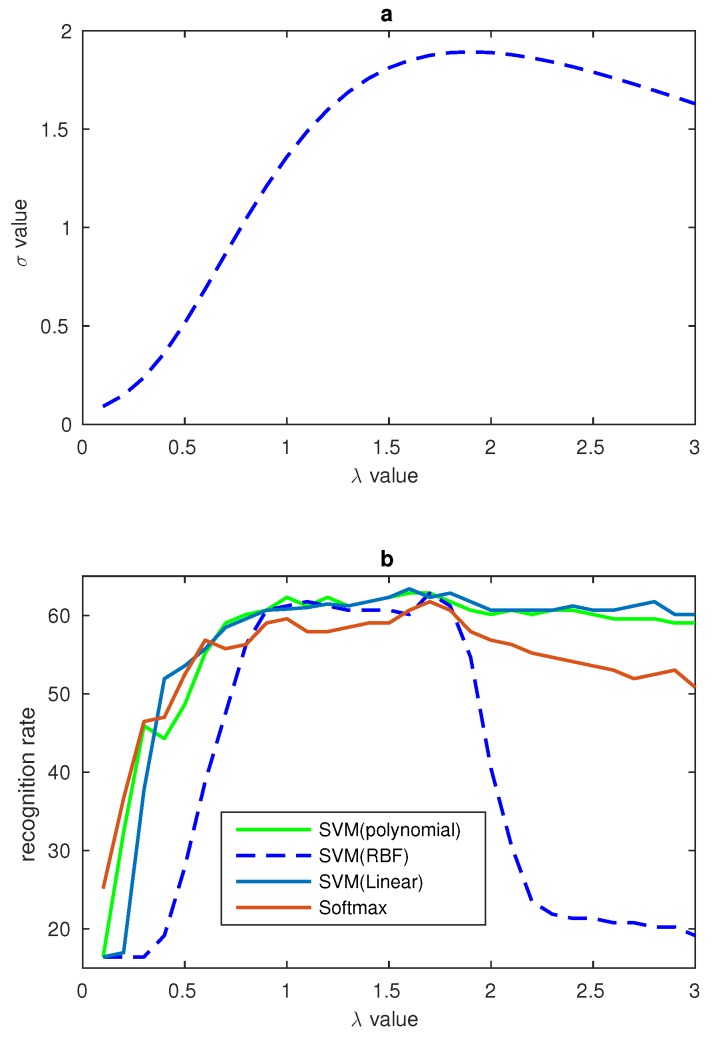
(**a**) Relationship between λ and σ on JAFFE database; (**b**) Relationship between λ and recognition rates from different classifiers on JAFFE database.

Principal Component Analysis (PCA) was invented in 1901 by Karl Pearson [28] as an analog of the principal axis theorem in mechanics, it was later independently developed by Harold Hotelling in the 1930s [29]. Principal component analysis (PCA) is a statistical procedure that uses an orthogonal transformation to convert a set of observations of possibly correlated variables into a set of values of linearly uncorrelated variables called principal components. PCA is an effective method to reduce the features dimension. There are many researchers using this method to reduce the features’ dimension. In our algorithm, the fusion features’ dimension is 11,636, which is really a very large number. In order to reduce the feature’s dimension, PCA is applied. The relationship between the recognition date and the number of the dimension under softmax classifier is shown in Figure 10. According to the experiment, the most appropriate dimension is 80.

There are many kinds of classifiers applied in the facial expression recognition research and the researchers apply different classifiers to evaluate their algorithms. These classifiers include SVM with polynomial [4,13], linear [2,4,13] and RBF [4,13] kernel, and softmax [10,15] is also utilized in this work. In order to evaluate the effectiveness of our proposed algorithm and compare with the same work in other literature, many classifiers are applied in our experimental part. In our algorithm, different classifiers are applied to recognize these fusion features the dimensions of which are reduced by PCA.

## 3. Database Processing

### 3.1. CK+ Database

The CK+ database [5] is an extended database of the CK [30] database which contains both male and female subjects. There are 593 sequences from 123 subjects in the CK+ database, but only 327 sequences are assigned to 7 labels. These 7 labels are anger (45), contempt (18), disgust (59), fear (25), happy (69), sad (28), surprise (83). The sequences show the variation in the images from neutral to the peak of the expressions. The different expressions have different numbers in the sequences. In particular, the images extended in 2010 have different pixels and two types of pixels, which are 640 × 490 and 640 × 480 in the database. In order to compare with other methods [2,3,14,27], our experiments use these 309 sequences in the 327 sequences without contempt. Similar to the method used in [2,3], the first image (the neutral) and the last three peak frames are chosen for training and testing. The ten-fold-validation method is applied in the experiments while the subjects are separated into 10 parts according to the ID of every subject. There are 106 subjects in the chosen database, so the subjects are distributed into 10 parts which have roughly equal image number and subject number.

### 3.2. JAFFE Database

The JAFFE database [31,32] consists of 213 images from 10 Japanese female subjects. Every subject has 3 or 4 examples of all the six basic expressions and also has a sample of neutral expression. In our experiment, 183 images are used to evaluate our algorithm.

## 4. Experiments

In this section the experimental setting and the details of our paper will be described. All comparison experiment ideas came from the second section of our paper and these experiments are applied to evaluate our methods and certify our algorithm’s correctness. Our experiments are executed on CK+ and JAFFE database and the results are also compared with the recognition rates in the related literature.

### 4.1. Salient Areas Definitude Method Validation

Th reasons why the salient areas rather than the whole faces are chosen in our algorithm have been introduced in Section 2. Experiments are designed to evaluate the performance of our salient areas’ definitive method. In addition, the LBP features are extracted from all of the aligned faces and the aligned salient areas to gain the contrast recognition rates. These results are shown in Table 3. In addition, the salient areas are separated from the raw images rather than the alignment faces according to specific landmarks among the 68 landmarks on the face. In Table 3, the 10-fold cross validation method is used to evaluate the performance of our method, and in this case only the LBP features are used in our recognition experiment.

For the purpose of distinguishing that whether using the salient areas can be more effective than the whole face alignment methods or not, the mouth areas are normalized to 60 × 30, the cheek areas are normalized to 30 × 60 while the eye areas are normalized to 20 × 90. LBP features are extracted from the small patches whose sizes are 15 × 10 and then all these features are concatenated together. LBP features are used to evaluate the performance of our algorithm and compare the result with other methods. The results are shown in Table 3. Several comparison experiments are designed, SVM and classifier are applied to evaluate our algorithm. Comparing LBP features extracted from the alignment areas with the features extracted from salient areas on alignment faces and the whole alignment faces, better recognition rates can be gained by our algorithm by using the SVM classifier. Polynomial, Linear, and RBF kernel SVM are used in our experiment and the SVM classifier is designed by Chih-Chung Chang and Chih-Jen Lin [33]. The gamma correction method is used to process the LBP features in our experiment. Compared with the experiment designed by Zhong et al. [3] and Liu et al. [2], according to the results in Table 3, our algorithm has a more precise recognition rate.

### 4.2. Gamma Correction of LBP Features

In Section 2.3, a method was proposed to process the LBP features and the relationship between the σ and gamma number λ was found. In order to evaluate the effects of gamma correction and verify the relationship between σ and gamma number λ some comparison experiments are designed in our paper. CK+ and JAFFE datasets are used in our experiments. In our experiments, the number of λ ranged from 0.1 to 3 and all the results are recorded. In order to show the relationship between σ, gamma correction number λ and the recognition rate clearly, some figures have been draw to display the trend of recognition rate and σ. In Figure 9 the σ is the sum of the four salient areas’ σ in CK+ database. In the figure, while λ is equal to 1 there is no gamma correction, and from the figure one can see that the biggest recognition rate and the biggest σ value all result from the value of λ near to 1.8. The relationship between σ and λ of JAFFE database are shown in Figure 10. These two figures show that our LBP correction method has good universality power. In addition, because there are fewer images in the JAFFE database, we can see that the curves in Figure 10 are not smooth enough, but their overall trends also correspond with the relationship in Figure 10. The performances of these experiments are shown in Table 4, and different classifiers are used to evaluate the universality of the gamma correction method. Table 4 shows that gamma correction has significantly improved the recognition rate and this proves that our method of using σ to find the proper λ can be applied in facial expression recognition work. In Table 4, 10-fold cross validation is applied on the CK+ database and the leave-one-person-out validation method is used on the JAFFE database.

In order to compare our algorithm with other research, we apply the same classifier and validation method as in the literature. Compared with the literature in Table 5, our experiment on the CK+ database has better results and this shows that our salient areas definitude methods and LBP correction method have fine performance.

### 4.3. Features Fusion, PCA and Recognition Rate Comparison

In our algorithm, LBP and HOG features are used to train the SVM and softmax classifiers and these features all are extracted from these salient areas. In order to gain a better result, LBP and HOG features are fused in our research. Using only the HOG feature, we obtain a 96.7 recognition rate and, using the fusion method, we obtain a better result, namely 98.3, which was reached on the CK+ database. In addition, a similar result has been obtained on the JAFFE database. Because using fusion features can lead to a better recognition result, fusion features are used in our algorithm.

**Figure 11:**
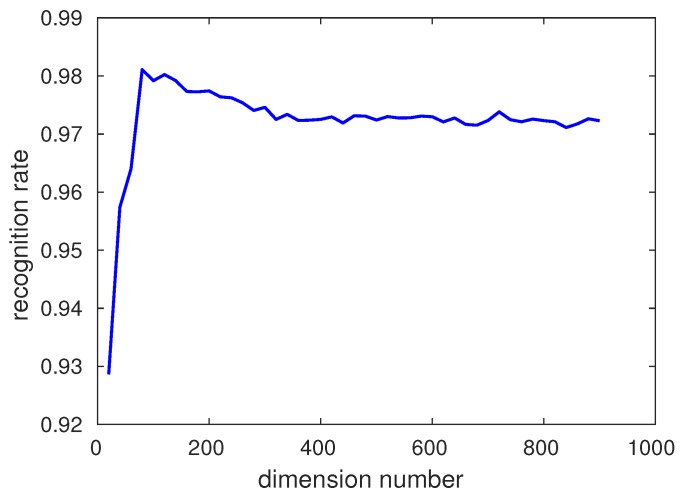
Relationship between Principal Component Analysis (PCA) dimension and recognition rate.

The full dimension of fusion features is 11,636, which is a very large number. In addition, huge feature dimension can pull in some noise and lead to overfitting. As for the PCA method, if the number of the features’ dimension is bigger than the images’ number the principal component number is 1. In order to gain the most appropriate number, the number of the dimension is changed from 10 to 1000 and by using this method, the PCA dimension can be chosen according to recognition rate. Because using the softmax classifier can obtain better recognition rate than the other classifiers, we use softmax to show the effects of PCA. The relationship between PCA number and recognition rate is displayed in Figure 11. The most appropriate PCA dimension number is chosen according to the recognition rate and the dimension number of the features put into softmax is 80 on CK+ database and a similar curve is obtained by JAFFE.

Until this step, the best recognition rate of 98.3 is gained under the 10-fold-cross validation method on the CK+ database. Therefore, to our knowledge, compared with the other methods in the literature, a state-of-the-art result has been obtained. Our result has been compared with other methods in the literature and the results are shown in Table 6. These four experiments all used deep networks while hand-crafted features are used in our algorithm. This explains that our algorithm has fine recognition ability by extracting features from the salient areas, correcting LBP features and fusing these features. In order to evaluate the adaptability of our algorithm, our algorithm also is applied on the JAFFE database. The results from other literature and our algorithm are shown in Table 7. The experiment shows that our algorithm has quite a good adaptability. Compared with the literature [13], our method can recognize about 5 more images than their method on average. In addition, compared with the literature [4], our features’ dimension on CK+ and JAFFE is 11,636 which is much less than 16,640, and this illustrates that our algorithm needs less time and memory to train and predict.

## 5. Discussion

More information about salient areas definitude methods is needed. Zhong et al. [3] designed a two-stage multi-task sparse learning algorithm to find the common and specific salient areas. LBP features rather than these pure data are used in this method, i.e., the LBP features are used to represent the information from the images. Liu et al. [2] built a convolutional layer and a max-pooling layer to learn the Micro-Action-Pattern representation which can depict local appearance variations caused by facial expressions. In addition, feature grouping was applied to find the salient areas. Compared with these two methods, our algorithm only uses the raw image data and there is no training procedure, but neutral faces are needed in our algorithm. For facial expressions, only partial areas in the face have changed, so the neutral faces can be used to calculate the correlation between neutral faces and specific expressions to localize the changed areas. Besides, the localiozation result can be more accurate when the changes are smaller. Furthermore, LBP features extracted from the small areas can also be used to compare and find the correlation. That is to say, by using these descriptors’ property, our algorithm can be used to localize the changed areas.

Making the features extracted from different subjects in one class have a similar value is the main reason why gamma correction can improve our recognition rate. On the surface, gamma correction has changed the display of LBP features, but in fact it has changed the value of LBP features. Different subjects have different ways of presenting the same expression so their LBP features have some difference. According to our experiments, although their LBP features’ values have some difference, their basic properties are similar. For instance, LBP features from the happy class have different values but these values are more different from those of the other classes. However, some subjects from different classes have similar LBP features and this is the reason why these algorithms cannot recognize the expressions. Our gamma correction method has shortened the distance in one class and this improves the recognition rate. The application of gamma correction on LBP features has had a positive effect on the recognition result and therefore some correction methods also can be applied on other features to shorten the distance in the same class to obtain a better recognition.

Although our algorithm has achieved a state-of-the-art recognition rate, there are some weakness in our method. Our algorithm selects these salient areas according to the landmarks on the faces, and if the landmarks are not accurate our recognition result will be influenced. Furthermore, if there is not enough image data, our gamma correction can not improve the recognition a lot. The performance of gamma correction on the JAFFE database shows these weaknesses. These are the main weaknesses of our algorithm.

## 6. Conclusions

The main contributions of this paper are summarized as follows: (1) A salient areas definitude method is proposed and the salient areas compared to neutral faces are found; (2) The idea of normalizing the salient areas to align the specific areas which express the different expressions is firstly proposed. This makes the salient areas of different subjects have the same size; (3) The gamma features correction method is firstly applied on the LBP features and this significantly improves the recognition result in our algorithm frameworks; (4) Fusion features are used in our framework, and by normalizing these features to the same scale, this significantly improves our recognition rate. By applying our algorithm framework, a state-of-the-art performance in the CK+ database under the 10-fold validation method using hand-crafted features has been achieved. In addition, a good result in the JAFFE database has also been obtained.

In the future, video data processing will be the focus of our research work and we will try to recognize facial expressions from real-time videos.

## Figures and Tables

**Table 1:** Patches numbers and LBP dimensions of salient areas.

Salient Areas	Forehead	Mouth	Left Cheek	Right Cheek
Piexls	20 × 90	40 × 60	60 × 30	60 × 30
Small patches number	12	16	12	12
LBP dimension	708	944	708	708
Total	**3068**			

**Table 2:** Patches numbers and Histogram of Oriented Gradient (HOG) dimensions of salient areas.

Salient Areas	Forehead	Mouth	Left Cheek	Right Cheek
Piexls	20 × 90	40 × 60	60 × 30	60 × 30
Small patches number	51	77	55	55
HOG dimension	1836	2772	1980	1980
Total	**8568**			

**Table 3:** Recognition rate on CK+ under different salient areas definitude methods.

Salient Areas Definitude Methods	Zhong 2012 [3] (MTSL)	Liu 2015 [2] (LBP)	Liu 2015 [2] (AUDN-GSL)	Proposed Method (with Gamma)	Proposed Method (without Gamma)
Classifer	SVM	SVM	SVM	SVM	SVM
Recognition rate	89.9	92.67	95.78	95.5	96.6

**Table 4:** Recognition rates on different classifiers with and without gamma correction.

	CK+		JAFFE	
	Gamma-LBP	LBP	Gamma-LBP	LBP
SVM(polynomial)	96.6	95.5	62.8	62.3
SVM(linear)	96.6	95.6	63.4	60.8
SVM(RBF)	96.0	87.1	62.8	61.2
Softmax	97.0	95.6	61.7	59.6

**Table 5:** Recognition rate on CK+ under LBP feature in different literature.

Methods	Zhong 2012 [3]	Shan 2009 [4]	Proposed Methods
Classifier	SVM	SVM	SVM
Validation Setting	10-Fold	10-Fold	10-Fold
Performance	89.9	95.1	96.6

**Table 6:** Recognition rate on CK+.

Literature	Liu 2014 [14]	Liu 2015 [2]	Jung 2015 [10]	Khorrami 2015 [15]	Proposed Algorithm
Method	BDBN	AUDN	DTAGN	Zero-biasCNN+AD	LBP+HOG
Validation Setting	10-Fold	10-Fold	10-Fold	10-Fold	10-Fold
Accuracy	96.7	95.785	96.94	98.3	98.3

**Table 7:** Recognition rate on JAFFE.

Literature	Shan 2009 [4]	Happy 2015 [13]	Proposed Algorithm	Proposed Algorithm	Proposed Algorithm
Classifier	SVM(RBF)	SVM(Linear)	SVM(Linear)	SVM(Linear)	Softmax
Validation Setting	10-Fold	5-Fold	5-Fold	10-Fold	10-Fold
Accuracy	81.0	87.43	87.6	89.6	90.0
